# Mechanisms Involving Ang II and MAPK/ERK1/2 Signaling Pathways Underlie Cardiac and Renal Alterations during Chronic Undernutrition

**DOI:** 10.1371/journal.pone.0100410

**Published:** 2014-07-01

**Authors:** Paulo A. Silva, Gustavo Monnerat-Cahli, Amaury Pereira-Acácio, Ricardo Luzardo, Luzia S. Sampaio, Marcia A. Luna-Leite, Lucienne S. Lara, Marcelo Einicker-Lamas, Rogério Panizzutti, Caroline Madeira, Leucio D. Vieira-Filho, Carmen Castro-Chaves, Valdilene S. Ribeiro, Ana D. O. Paixão, Emiliano Medei, Adalberto Vieyra

**Affiliations:** 1 Carlos Chagas Filho Institute of Biophysics, Federal University of Rio de Janeiro, Rio de Janeiro, Brazil; 2 National Institute of Science and Technology for Structural Biology and Bioimaging, Rio de Janeiro, Brazil; 3 Institute of Biomedical Sciences, Federal University of Rio de Janeiro, Rio de Janeiro, Brazil; 4 Department of Physiology and Pharmacology, Federal University of Pernambuco, Recife, Brazil; Pennington Biomedical Research Center, United States of America

## Abstract

**Background:**

Several studies have correlated protein restriction associated with other nutritional deficiencies with the development of cardiovascular and renal diseases. The driving hypothesis for this study was that Ang II signaling pathways in the heart and kidney are affected by chronic protein, mineral and vitamin restriction.

**Methodology/Principal Findings:**

Wistar rats aged 90 days were fed from weaning with either a control or a deficient diet that mimics those used in impoverished regions worldwide. Such restriction simultaneously increased ouabain-insensitive Na^+^-ATPase and decreased (Na^+^+K^+^)ATPase activity in the same proportion in cardiomyocytes and proximal tubule cells. Type 1 angiotensin II receptor (AT_1_R) was downregulated by that restriction in both organs, whereas AT_2_R decreased only in the kidney. The PKC/PKA ratio increased in both tissues and returned to normal values in rats receiving Losartan daily from weaning. Inhibition of the MAPK pathway restored Na^+^-ATPase activity in both organs. The undernourished rats presented expanded plasma volume, increased heart rate, cardiac hypertrophy, and elevated systolic pressure, which also returned to control levels with Losartan. Such restriction led to electrical cardiac remodeling represented by prolonged ventricular repolarization parameters, induced triggered activity, early after-depolarization and delayed after-depolarization, which were also prevented by Losartan.

**Conclusion/Significance:**

The mechanisms responsible for these alterations are underpinned by an imbalance in the PKC- and PKA-mediated pathways, with participation of angiotensin receptors and by activation of the MAPK/ERK1/2 pathway. These cellular and molecular alterations culminate in cardiac electric remodeling and in the onset of hypertension in adulthood.

## Introduction

Chronic undernutrition (with severe protein restriction) is a worldwide public health problem, especially in developing countries [1], where young people are particularly vulnerable to the consequences of food restriction, including the development of different diseases in later life (adulthood) [2]. Some developmental changes lead to the onset of hypertension [3], heart disease [4], and kidney disease [5].

Many experimental studies designed to elucidate the mechanisms by which chronic undernutrition provokes functional and morphological alterations in various human systems – including cardiac and renal Na^+^ transport systems – have focused on the prenatal and lactation periods [6]–[8]. However, chronic undernutrition provoked by protein restriction associated with other dietary deficiencies is not confined to early life; it is a widespread lifelong condition frequently imposed from conception, persisting through growth and development into adult life [9].

The cardiovascular system and the kidneys are special targets of protein restriction (for classical reports concerning clinical and experimental data see refs. [10], [11]). As an example of combined vascular and renal alterations, early studies demonstrated that an isocaloric-hypoproteic diet in rats led to hemodynamic alterations, in which increased afferent and efferent arteriolar resistance was responsible for a decline in both glomerular filtration rate and renal blood flow [12]. In humans, some epidemiological studies have indicated a low incidence of hypertension under conditions of chronic undernutrition [13], [14]; but the opposite trend was found in other studies [15]. These differences could be ascribed to the differing proportions of nutrients in deficient diets.

Common mechanisms seem to underlie cardiovascular and renal alterations, including those that share common modifications in the reactivity and participation of the systemic and local renin angiotensin system (RAS). Cardiovascular and renal changes occurring over short periods of protein restriction in adult rats correlate with altered responsiveness to angiotensin II (Ang II) [10], [16], but no studies seem to have focused on the relationships among different transport/signaling systems that are crucial for cardiac and renal functions.

The driving hypothesis for the present work was that Ang II-signaling pathways linked to Na^+^ pumps in heart and kidney are affected by chronic administration of a low-protein multideficient diet, promoting electric cardiac remodeling and other renovascular alterations, which could culminate in the onset of hypertension. To test the hypothesis that angiotensin II type 1 receptors (AT_1_R) are involved in the cardiorenal alterations, we analyzed the effect of simultaneous administration of the receptor blocker Losartan (Los). We used a diet prepared according to data from food consumption surveys in different geographic zones of Northeast Brazil (the Basic Regional Diet/BRD) [17]. This diet mimics other deficient diets that are consumed in many parts of the world.

## Materials and Methods

### Ethical considerations

All experimental procedures were approved by the Committee for Ethics in Animal Experimentation of the Federal University of Rio de Janeiro (protocol N° IBCCF 104), and were carried out in accordance with the Committee's guidelines, which follow the Uniform Requirements for Manuscripts Submitted to Biomedical Journals.

### Animals and experimental groups

The experimental groups consisted of male Wistar rats (Biocampo and Fiocruz) that were kept at 23±2°C in a 12 h/12 h light/dark cycle. The animals were divided into four groups: (i) control (CTR) rats, with free access to standard chow (Purina Agribands) and water from weaning (21 days after birth) to 90 days of age; (ii) rats chronically subjected to the basic regional diet (BRD), with free access to the deficient diet and water from weaning to 90 days of age; (iii) CTR Los, control rats receiving Los (Merck) by gavage (30 mg/kg body mass) from weaning (21 days after birth) to 90 days of age; and (iv) BRD Los, BRD rats receiving the same treatment with Los. Several series of successive breedings using 3-month old female rats (∼250 g) and male rats in a 3∶1 ratio gave the number of pups from different litters required for isolation of adequate membrane preparations, and for the *in vivo* experiments (chronic administration of the AT_1_ receptor antagonist, Los, measurement of plasma volume, arterial pressure and heart recording, food and water intake assessment, blood collection for plasma amino acid analyses). The female pups were humanely killed by decapitation in accordance with the guidelines mentioned above. All series gave consistent biochemical/immunochemical results with no differences within the same class of experiment. Litter effects were avoided by randomly assigning male offspring from the same litters to the four conditions to which they were subjected after weaning.

### Number of animals

At the start, the number of rats in a typical experimental set was 10–15 for the CTR and CTR Los groups, and 20–25 for the BRD and BRD Los groups. The larger number of initially undernourished rats compared with the normonourished groups was due to the shorter lifespan of the former (at 90 days of age) (some rats from the undernourished groups do not live 90 days). These runs were repeated 4 times. For the *in vivo* experiments, the number of rats from each group (see the corresponding figure legends) includes animals from the different runs. For arterial pressure measurements, the number of rats in each group (n = 7) corresponds to recordings obtained in 6 animals from 3 different litters (2 for each) and 1 animal (from 1 run), due to the special conditions required to obtain accurate readings quickly and in the same time of the day (between 02:00 and 06:00 pm).

Five rats from the CTR and CTR Los groups and 7 rats from the BRD and BRD Los groups were used to isolate the plasma membranes from cardiomyocytes and proximal tubule cells, and to obtain pooled membrane preparations for the *in vitro* experiments. The number of pooled samples ranged from 5 to 7. Due to the limited amount of protein and the wide variation in the experiments, a larger number of experiments were carried out when the determinations showed high variability, as in the case of PKA. The number of membrane preparations used in each class of experiment is given in the corresponding figure and table legends. For the electrophysiological studies, small fragments of the left ventricle were removed isolated from different rats prior homogenization of the rest of the organ.

### Diets

The deficient diet contained the following ingredients (g/g%): beans, 18.3; manioc flour, 64.8; jerked meat, 3.7; and sweet potatoes, 12.8 [17]. The ingredients were cooked, dehydrated at 60°C and pulverized. This diet provides the following percentage composition (g/g%): protein, 8; carbohydrate, 69; lipid, 0.8; Na^+^, 0.2; fiber, 8. Besides the lower percent, the diet is deficient in the quality of proteins, since >90% is provided by beans and <10% by meat. Even though the adequacy of energy supply is slightly higher (∼316 kcal/100 g dry weight) than the control diet (∼280 kcal/100 g), only a small fraction is provided by fats, corresponding to ∼1% of the dry mass in contrast to ∼9% in the control diet. Most of the calories come from carbohydrates (sweet potatoes and manioc flour). BRD has only ∼30% of the total mineral salts present in the control diet, with important differences regarding its specific components. Ca^2+^ (0.04 g/g%), K^+^ (0.3 g/g%), and iron (0.007 g/g%) contents are much below those in the control diet (1.8, 0.9 and 0.018, respectively) [18]. Na^+^ oscillates in the control range (0.2–0.4 g/g% according to repeated measurements in samples taken at random). Several vitamins (ascorbic acid, retinol, biotin, thiamin, riboflavin, niacin, para-aminobenzoic acid, pyridoxine, inositol, cyanocobalamine, choline) are extremely low, as previously calculated by Teodósio et al. [17] from the vitamin content of the components of BRD [19]. The nutrient contents in the control diet (regular chow diet from Purina Agriband) were (g/g%): protein, 23; carbohydrate, 50; lipid, 9; and Na^+^, 0.3, which gives a total of 280 kcal/100 g dry weight. This was supplemented with vitamins to meet AIN-93G requirements [20], whereas the deficient diet was not supplemented.

### Plasma volume, systolic arterial pressure, heart rate and plasma amino acids, determinations

Plasma volume, arterial pressure and heart rate were assessed at 90 days. Plasma volume was assessed using Evans Blue Dye. After the rats had been anesthetized with pentobarbital (60 mg/kg), a femoral artery was catheterized and a 1 ml basal blood sample was collected. Evans Blue dye (Sigma-Aldrich; 0.1% in 150 mM NaCl) was then administered (100 µg/100 g body weight) through the catheter. The catheter was filled with physiological saline to push all the injected dye into the animal. After 7.5 min, the NaCl solution inside the catheter was discarded and 1 ml of blood was collected in a heparinized syringe. The concentration of the Evans Blue dye was measured spectrophotometrically at 610 nm and compared to a standard curve obtained with known dilutions of the dye in the basal plasma (before dye injection).

Systolic arterial pressure and heart rate were measured using the tail cuff method, which allows repeated and reliable non-invasive measurements to be made over a short period in conscious animals [21], [22]. A Letica LE 5000 pletismograph was used (Panlab). Animals from different litters (see above) were acclimated by placing them for 10–15 min each day for a week in the chamber at 30–32°C before taking recorded measurements. On the day of measurements, the rats were held for 10 min to carefully checked that they had stopped moving before starting the recording. If any movement was detected, another attempt was made after a further 10 min period. Three successive determinations were made for each animal and the procedure was repeated on 3 consecutive days. A typical data acquisition lasted ∼30 min. Two criteria were used to assess that the readings were correct: (i) the intra-assay coefficient of variation among the 3 different determinations, which were always <5% (ranging from 2.3 for CTR to 3.4% for BRD); (ii) the stability of heart rhythm after the 10 min preparation period.

Blood samples for determining plasma amino acids were collected at 85 days of age and EDTA was added. After separation of the cells by centrifugation, the plasma was analyzed by high performance liquid chromatography (HPLC) as described in [23], [24].

### Food and water intake

Food and water intake was assessed in metabolic cages, as previously described [22], [25].

### ECG and action potentials recording

Electrocardiograms were recorded from anesthetized animals (Xylazine and Ketamine, 15 and 80 mg/kg ip, respectively). Electrodes were positioned in DI derivation and connected by flexible cables to a differential AC amplifier (model 1700, A-M Systems), with signals low-pass filtered at 1 kHz and digitized at a 2–10 kHz sample rate by a 16-bit A/D converter (Minidigi 1-D, Axon Instruments) using Axoscope 9.0 software (Axon Instruments). Data were stored in a PC for offline processing.

Both right and left endocardial ventricle preparations were used to assess the action potential profile [26]. Muscle strips (approximately 0.5 cm×0.5 cm×0.1 cm) were obtained and pinned in order to expose the endocardial side above the bottom of a tissue bath. The preparations were superfused with an oxygenated (95% O_2_, 5% CO_2_) Tyrode's solution containing (in mM) 150.8 NaCl, 5.4 KCl, 1.8 CaCl_2_, 1.0 MgCl_2_, 11.0 D-glucose, 10.0 HEPES (pH 7.4 adjusted with NaOH at 37±0.5°C) at a flow of 5 ml/min (Gilson Miniplus 3). The tissue was stimulated at four different basic cycle lengths (BCL) (1000, 800, 500 and 300 ms) using field stimulation. The transmembrane potential was recorded using glass microelectrodes (10–40 MΩ DC resistance) filled with 2.7 M KCl connected to a high input impedance microelectrode amplifier (MEZ7200, Nihon Kohden). Amplified signals were digitized (1440 digidata A/D interface and Axotape software, Axon Instrument, Inc.) and stored in a personal computer for later analysis using the software Clampfit 10.2 (Axon Instrument, Inc.). The following action potential parameters were analyzed: resting membrane potential (RMP), action potential amplitude (APA) and action potential duration at 90% (APD_90_), 50% (APD_50_) and 30% (APD_30_) repolarization. The Maximum Negative Slope (MaxNegSlope) was calculated by the steepest downhill slope starting 5 ms after the peak using a linear regression during a window of 4 ms. The AP triangulation was calculated by subtracting APD_40_ from APD_90_. To assess the presence of arrhythmic events, a 10 beat train pulse followed by a pause was applied at three different BCLs (200, 150 and 100 ms).

### Isolation of cardiomyocyte plasma membranes

Plasma membranes from cardiomyocytes were prepared by differential centrifugation as in [27].The hearts were removed together with the kidneys after decapitation of the rats at 90 days of age, placed on ice and carefully dissected to obtain the left ventricle and the septum, which were first minced into small fragments to obtain, with slight modifications, a membrane preparation that was previously shown to be adequate for assays of (Na^+^+K^+^)ATPase activity with ^3^H-ouabain and immunoassays for (Na^+^+K^+^)ATPase [27]. Briefly, the fragments obtained from 5–7 hearts from each experimental group (see “number of animals” above) were suspended in an isotonic solution containing 250 mM sucrose, 1 mM imidazole (pH adjusted to 7.6 with Tris) and 1 mM EDTA to obtain pooled preparations. These were mechanically homogenized at 4°C using a Potter Elvejhem homogenizer fitted with a Teflon pestle (five periods of 1 min at 1,700 rpm). The preparation was centrifuged at 1,669×*g* and the resulting supernatant was centrifuged again at 115,000×*g*; the final sediment was suspended in 250 mM sucrose and stored under liquid N_2_. Five to 7 pooled cardiac membrane preparations were thus obtained for biochemical determinations (see below). The protein concentration was measured by the Folin method [28]. The small pieces of left ventricle taken for electrophysiological measurements were homogenized and used immediately.

### Isolation of proximal tubule cell membranes

Plasma membranes from proximal tubule cells were also prepared by differential centrifugation as in [29]. The kidneys were placed in an isotonic solution containing 250 mM sucrose, 10 mM Hepes-Tris (pH 7.4), 2 mM EDTA and 0.15 mg/ml trypsin inhibitor type II-S (Sigma-Aldrich) (1 g tissue/4 ml solution). Membranes from different rats (see “number of animals” above) were prepared from the outer region of the cortex (*cortex corticis*) as described elsewhere [30], where the predominant cell population is proximal tubule cells [31]. Controls for enrichment with basolateral membranes (3–4 fold with respect to the total homogenate using (Na^+^+K^+^)ATPase as a marker) and for minimal residual contamination with intracellular membranes and cytosol were as described in [29], [30]. No attempt at further enrichment was made in this case, as the (Na^+^+K^+^)ATPase and the ouabain-resistant Na^+^-ATPase are exclusively located in the basolateral membranes of epithelial cells [32], and a low yield of purified basolateral membranes was obtained using the Percoll gradient method with the minimum number of animals recommended by the Committee for Ethics in Animal Experimentation. The plasma membrane fraction was stored under liquid N_2_. The protein concentration was also measured by the Folin reagent method [28].

### Measurement of ouabain-insensitive Na^+^-ATPase and of (Na^+^+K^+^)ATPase activities

Ouabain-resistant, furosemide-sensitive Na^+^-ATPase and (Na^+^+K^+^)ATPase activities were measured as in [8], [22]. Ouabain-resistant, furosemide-sensitive Na^+^-ATPase activity was measured as the difference in ^32^P_i_ released from (γ-^32^P)ATP in the absence and presence of 2 mM furosemide (Sigma-Aldrich) (with 2 mM ouabain throughout), as described elsewhere [8], [22], [30]. (γ-^32^P)ATP was prepared as per Maia et al. [33], using radioactive orthophosphate (^32^P_i_) purchased from the São Paulo Institute of Nuclear and Energetic Research. The cardiac and renal membranes (0.2 mg protein/ml) were also preincubated with ouabain for 10 min at 37°C in a medium containing 20 mM Hepes-Tris (pH 7.0), 10 mM MgCl_2_ and 120 mM NaCl. The reaction was started by adding (γ-^32^P)ATP (5 mM, specific activity ∼1 µCi/µmol; final concentration). After 10 min at 37°C, the reaction was stopped with charcoal. The released ^32^P_i_ was quantified by liquid scintillation counting in an aliquot of the supernatant obtained after centrifugation of the charcoal suspension (1,500×*g* for 5 min).

(Na^+^+K^+^)ATPase activity was determined by measuring P_i_ release from ATP (Sigma-Aldrich) in the absence or presence of 2 mM ouabain (Sigma-Aldrich) as previously described [8], [22], [30]. The cardiac or renal membranes (0.05 mg protein/ml) were preincubated with ouabain for 10 min at 37°C in a medium containing 50 mM Bis-Tris-propane (pH 7.4), 0.2 mM EDTA, 5 mM MgCl_2_ and 120 mM NaCl. The reaction was started by adding a mixture of KCl and ATP (20 and 5 mM, respectively; final concentrations), and stopped after 10 min with 2 volumes of activated charcoal in 0.1 M HCl. The released P_i_ was quantified colorimetrically [34].

To investigate the possible involvement of the MAPK/ERK pathway in modulating the ouabain-insensitive Na^+^-ATPase activity in cardiac and renal membranes of undernourished rats, a series of assays were performed as above using 30 µM PD098059 (InvivoGen), a selective inhibitor of MAPK [35], to prevent phosphorylation of ERK1/2. The membranes were preincubated with the inhibitor for 10 min before addition of ATP.

### SDS-PAGE and immunoblotting

SDS-PAGE and imunoblotting for AT_1_R, AT_2_R, ERK1 and phospho-ERK1/2 were carried out as in [8], [36] using the specific antibodies. Briefly, the proteins of renal proximal tubule cells and cardiomyocytes were separated on 10% SDS-PAGE [37] and transferred to nitrocellulose membranes. Non-specific binding was prevented by incubating the membranes with 5% non-fat milk in Tris-buffered NaCl (TBS, pH 7.6) for 1 h. The membranes were probed with the corresponding primary antibodies against: AT_1_R or AT_2_R (1∶500, Santa Cruz Biotechnology in both cases); ERK1 (1∶1,000) or phospho-ERK1/2 (1∶500) (Cell Signaling in both cases); β-actin (1∶5,000, Sigma-Aldrich) for 1 h at room temperature with gentle stirring. Then, they were washed three times with TBS containing 0.1% Tween 20 (TBS-T), exposed to the secondary fluorescent antibodies (anti-rabbit, Li-Cor, IRDye 680RD, 1∶20,000 for AT_1_R, AT_2_R, ERK1 and phospho-ERK1/2; anti-mouse, Li-Cor, IRDye 800CW, 1∶20,000 for β-actin),and washed again. Immunoreactivity was detected using the Odyssey system (Li-Cor) for infrared imaging recording, and the band intensities quantified using Scion Image software. The β-actin immunosignal from the corresponding lane was used to normalize the immunosignals of AT_1_R, AT_2_R, ERK1 and p-ERK1/2 for protein loading. Duplicates for each analyzed protein – from heart or kidney membranes – corresponding to the four experimental conditions (CTR, BRD, CTR Los and BRD Los) were analyzed in the same gel, and the results were expressed as a percentage of the corresponding CTR value (taken as 100%). Each of these assays was repeated using different preparations (see “n” values in the corresponding figure legends). The Scion Image software was used to quantify the band intensities. In preliminary experiments, the nitrocellulose membranes were stained with *Ponceau Red* to assess protein loading.

**Figure 1:**
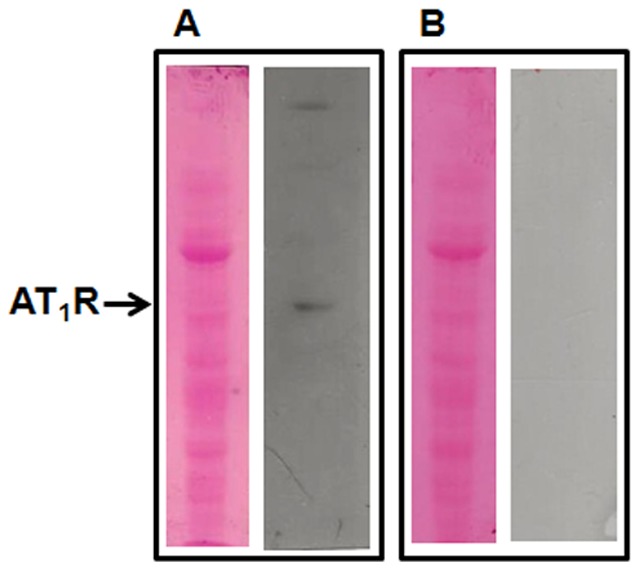
Specificity of the Ang II receptor antibodies. Electrophoresis of renal membranes was carried out as described in the Materials and Methods section, using 80 µg of total protein. The specificity of the antibodies was confirmed by a preadsorption experiment using full-length human Ang II type 2 receptor recombinant protein as a matrix. The samples were incubated for 72 h at 4°C with gentle stirring and then centrifuged at 18,000×*g* for 1 min. The secondary antibody was a polyclonal anti-rabbit (NIF824, GE; 1∶2,500). See additional details in the Materials and Methods section. A: Ponceau red-stained nitrocellulose membrane (left) and immunosignal obtained after incubation of the AT_1_R antibody with the full-length AT_2_R recombinant protein (right). B: Ponceau red-stained nitrocellulose membrane (left) with no signal when the AT_2_R antibody was preadsorbed on to the full-length AT_2_R recombinant protein (right).

Herrera et al. [38] demonstrated that several anti-Ang II receptors antibodies available from commercial sources display nonspecific binding in kidney and in other tissues. In the present work, the specificities of the primary antibodies against AT_1_R and AT_2_R were confirmed in the present study by preadsorption experiments using a matrix of human Ang II type 2 receptor recombinant full-length protein (amino acids 1–363, ab157871, Abcam), as in [39] for the anti-renin antibody. A three-fold mass of the immunizing peptide was incubated with the antibodies: (i) sc1173 Santa Cruz, anti-AT_1_R raised against a peptide mapping an N-terminal extracellular domain of AT_1_R (human); or (ii) sc-9040 Santa Cruz, anti-AT_2_R raised against amino acids 221–363 of AT_2_R (human). After centrifugation and dilution of the supernatants (1∶500), immunostaining demonstrated the presence of the AT_1_R band in renal membranes using the AT_1_R-containing solution, whereas no signal appeared when the AT_2_R-containing solution was used (Figure 1). These antibodies against AT_1_R and AT_2_R were those used in the immunoassays described above.

### Protein kinase C (PKC) and cyclic AMP-dependent protein kinase (PKA) activities

The activities of PKC and PKA were measured as in [8], [36].The activities of PKC and PKA associated with the isolated membranes of renal and cardiac origin were measured by incorporation of the γ-phosphoryl group of (γ-^32^P)ATP into histone in the absence and presence of their respective inhibitors: 10 nM calphostin C (Calbiochem) for PKC, 10 nM PKAi_(5–24)_ peptide (Sigma-Aldrich) for PKA [8], [30], [36]. The reaction was started by adding (γ-^32^P)ATP (10 µM; specific activity ∼4.5 µCi/nmol) to the reaction medium (0.1 ml) containing 20 mM Hepes-Tris (pH 7.0), 4 mM MgCl_2_, 1.5 mg/ml histone 2S (Sigma-Aldrich) and 0.7 mg/ml membrane protein. After 2 min, the reaction was stopped by adding 0.1 ml 40% (w/v) TCA and the samples were immediately placed on ice. After intense stirring, an aliquot of 0.1 ml was filtered through a Millipore filter (0.45 µm pore size) and successively washed with ice-cold 20% (w/v) TCA and 0.1 M phosphate buffer (pH 7.0). The radioactivity was quantified in a liquid scintillation counter.

### Statistical analysis

The data are shown as mean values ± SEM. The differences between the groups were analyzed by one-way ANOVA followed by a Tukey test or by one-way ANOVA followed by a Bonferroni test for selected pairs. Intra-assay coefficients of variation ((standard deviation/mean of 3 determinations)×100) per run were calculated for the arterial pressure recordings (see above). Intra-assay coefficients of variation for the immunosignals corresponding to each protein (AT_1_R, AT_2_R, ERK1 and phospho-ERK1/2 in the four experimental conditions), which were detected using the Odyssey system, were always less than 10% (see above).

## Results

### General data

**Figure 2:**
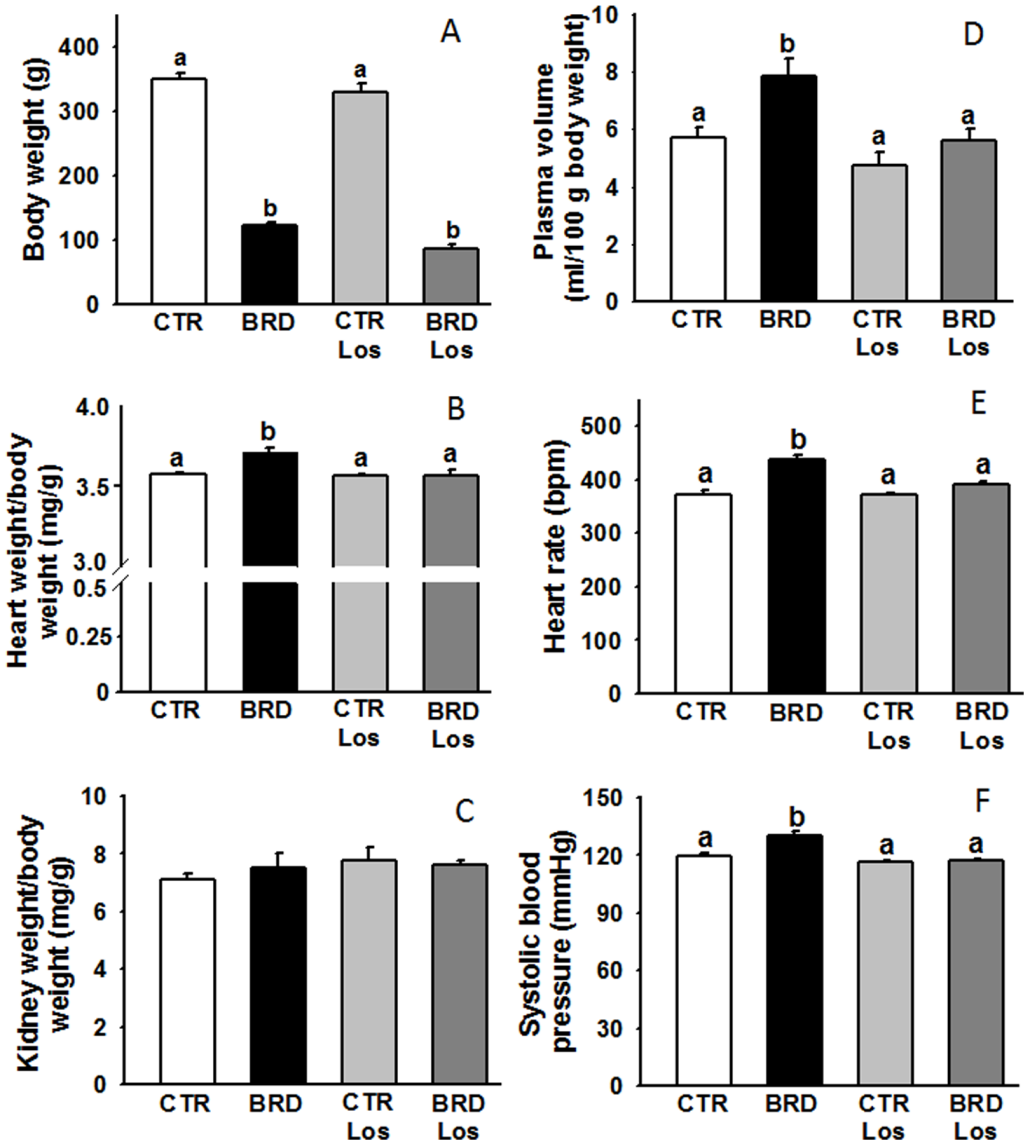
Alterations in body weight, cardiac index and renal index (A–C), and expanded plasma volume, accelerated heart rate and increased systolic pressure (D–F), in rats subjected to protein restriction (aged 90 days). The animal groups were: control (CTR); fed with the deficient diet after weaning (BRD); control receiving Losartan (CTR Los); and BRD receiving Losartan (BRD Los) (A–C, n = 7; D, n = 13 in CTR and CTR Los groups; n = 5 in BRD and BRD Los groups; E, n = 23 in CTR and CTR Los groups; n = 8 in BRD and BRD Los groups; F, n = 7 in all groups). Histograms show mean ± SEM. Different lower-case letters above the bars indicate statistically significant differences in mean values within the corresponding panel (P<0.05).

**Figure 3:**
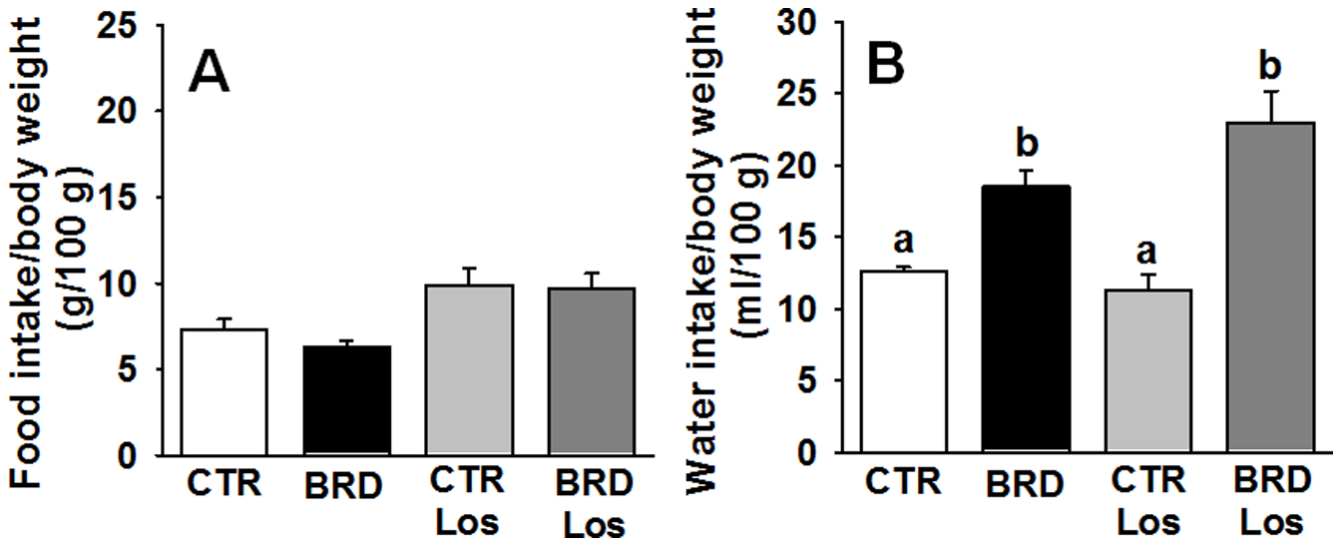
Food and water intake. Food and water ingestion was recorded one day before sacrifice at 90 days of age at the end of a 24 h period. The animal groups were those described in the legend to Figure 2. The rats were maintained in individual metabolic cages in the same conditions of light and temperature described in the Methods section of the main text. Simultaneous recording of body weight at the end of the period allowed correction of the data, as shown on the *abscissa*e. Different lowercase letters above the bars indicate statistically different mean values in panel B (P<0.05; n = 5).

Dietary restriction led to an accentuated decrease in the rate of body weight gain in both the BRD and BRD Los groups compared with the CTR- and CTR Los-matched groups. The lighter body weight at 90 days (Figure 2A) was accompanied by a significant, though small, increase in the heart index of the BRD rats (Figure 2B), which was prevented in the group treated with Los (BRD Los). The decrease in kidney weight paralleled the reduction in body weight and Los had no observable effect (Figure 2C). Also as expected, the same profile was seen when plasma volume, heart rate and systolic blood pressure were investigated: all increased in the BRD group (Figure 2D–F). Administration of Los to rats that had received the deficient diet restored all the altered values to the levels found in the control group. Food and water intake is presented in Figure 3. There was no difference in food intake among the four groups; but water intake was significantly increased in BRD rats. The AT_1_R antagonist Los did not influence this last parameter in both groups.

**Figure 4:**
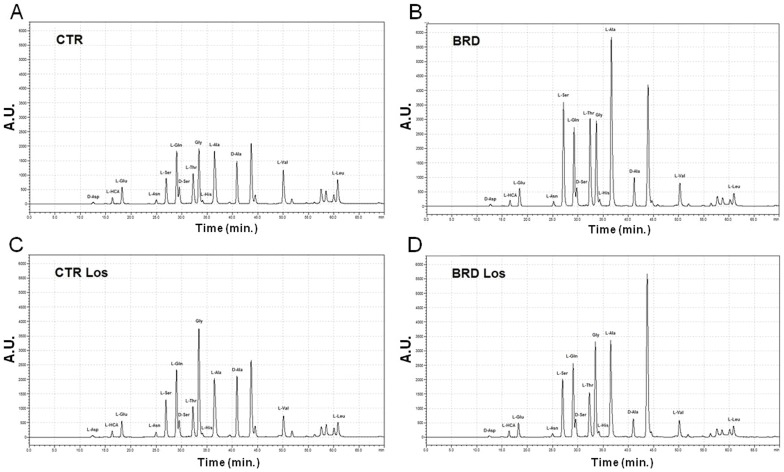
Representative plasma amino acids analysis. The plasma was analyzed by high performance liquid chromatography (HPLC) as described in Materials and Methods. Abbreviations of the experimental groups are those defined in the legend to Figure 2. The peaks were identified using individual amino acid standards that were run immediately after the plasma samples. Quantification and statistical analysis of the peaks are presented in Figures 5 and 6.

**Figure 5:**
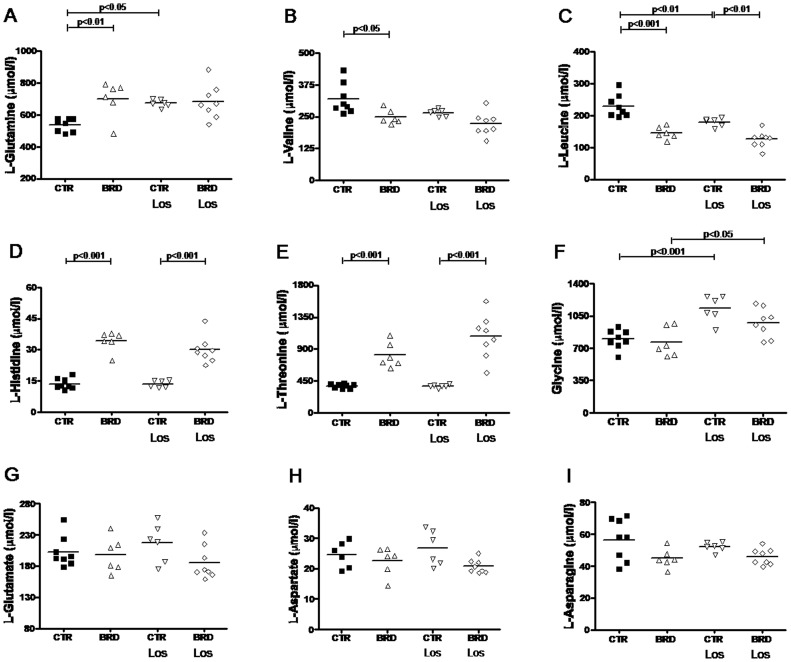
Changes in the plasma levels of L-amino acids and glycine. Panels show values for each animal. Horizontal lines represent mean values (n = 5–8 blood samples from different rats of each group). Statistical differences were assessed by one-way ANOVA followed by Bonferroni adjustment for CTR vs. BRD, CTR vs. CTR Los, BRD vs. BRD Los, and CTR Los vs. BRD Los, as indicated.

**Figure 6:**
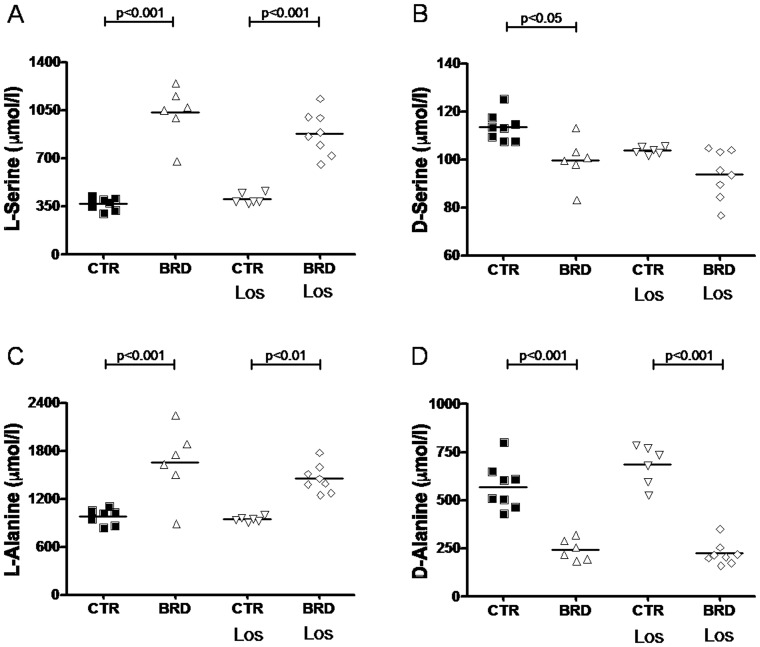
BRD induces increase in plasma levels of L-Serine and L-Alanine with simultaneous decrease in D-Serine and D-Alanine. Panels show values for each animal. Horizontal lines represent mean values (n = 6–8 blood samples from different rats of each group). Statistical differences were assessed by one-way ANOVA followed by Bonferroni adjustment for CTR vs. BRD, CTR vs. CTR Los, BRD vs. BRD Los, and CTR Los vs. BRD Los, as indicated.

Besides its accentuated lower protein level, the poor quality of the proteins (90% from beans, only 10% from meat) reflects on the amino acid content, with low levels of most essential and non-essential amino acids [17]. Thus, it was hypothesized that plasma amino acids in the BRD group could have suffered from quantitative/qualitative alterations. Quantification of aminograms shown (for a representative aminogram see Figure 4) corroborates this hypothesis; changes in dietary amino acid content were reflected in the plasma amino acids of the BRD group with an unexpected profile (Figures 5 and 6). BRD rats presented with increased levels of L-serine, L-Glutamine, L-Threonine, L-Histidine, L-Alanine and decreased levels of L-Valine and L-Leucine. Also interesting was the decrease in D-Serine and D-Alanine (Figure 6).

### Cardiac electrical remodeling and increased risk of arrhythmias in the BRD group

**Figure 7:**
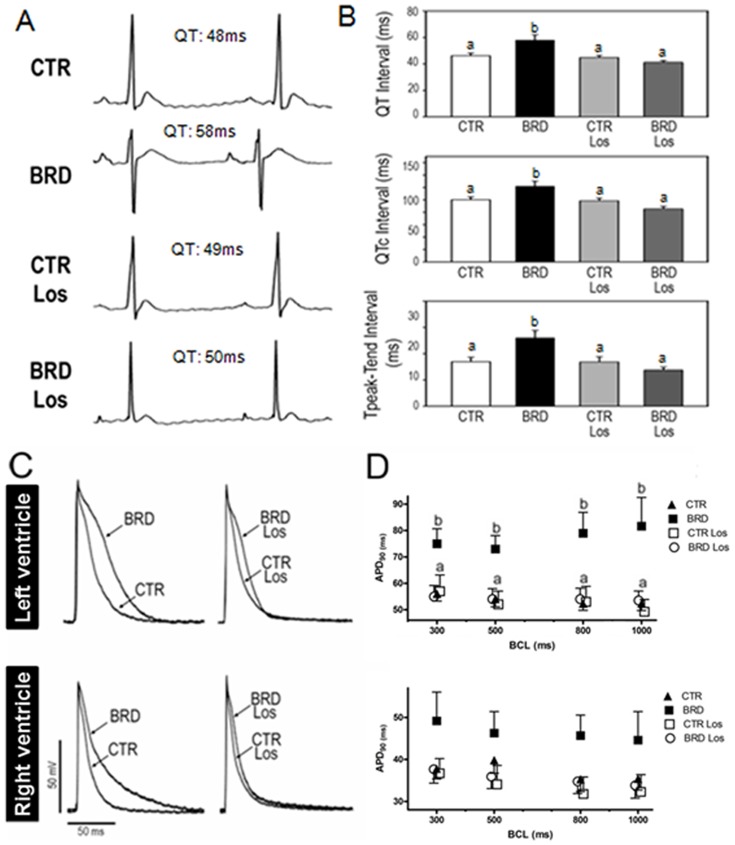
BRD induced longer QT, QTc, Tpeak-Tend and action potential duration. The animal groups were as described for Figure 2. (A) Representative traces of electrocardiograms show longer QT in the BRD group. (B) BRD induced ventricular repolarization disturbances, as summarized in the bar graph. Histograms show mean ± SEM. (C) Representative traces show longer left, but not right, ventricular action potential in BRD. (D) Longer ventricular action potential duration at 90% repolarization (APD_90_) in the BRD group under different basic cycle lengths (BCL), as shown on the abscissae (panels B and D, n = 6–10). Different lowercase letters above the bars indicate statistically different mean values within the corresponding panel (P<0.05).

The next step in understanding the effect of chronic protein restriction associated with deficiency of other nutrients was to study the cardiac electric activity, which could be associated with structural remodeling (higher heart index). The BRD group presented an expressive ventricular repolarization dysfunction, as indicated by a significantly longer QT interval than in the CTR group (Figure 7A and upper panel in Figure 7B). When this prolongation was corrected by Bazett's formula, no dependence on the heart rate could be seen (QTc, Figure 7B, middle panel). In addition, the Tpeak-Tend interval in the BRD group was longer than in the CTR group (Figure 7B, lower panel). Like the cardiovascular parameters (Figure 2D–E), all the abnormal ventricular repolarization parameters in ECG records regained normal profiles after Los treatment. The Los group revealed no difference in ECG tracings from the CTR group (Figure 7B), as expected from the hypothesis of a selective effect of chronic undernutrition on the AT_1_R-linked pathway.

The next step was to investigate whether the *in vivo* repolarization profile demonstrated by ECG correlated with the cardiac action potential, raising the possibility that BRD could affect the left ventricle selectively. The two aspects of this hypothesis were investigated by performing the experiments depicted in Figure 7C. The cardiac action potential (AP) tracing revealed that BRD provoked prolongation in the left but not the right ventricular tissue, and this was prevented by Los (Figure 7C). Figure 7D indicates that the longer AP duration at 90% of repolarization (APD_90_) in the BRD group was not dependent on stimulation frequency. This picture, intended to elucidate the electric parameters affected by BRD, was completed by the observation that proarrythmic markers, maximal negative slope (MaxNegSlope), and triangulation in AP were significantly different in BRD from the other three groups; however, no differences were observed among groups in the other AP parameters analyzed (Tables 1 and 2).

**Table 1:** Action potential parameters recorded in left endocardial ventricle preparation.

Variables	Groups			
	CTR	BRD	CTR Los	BRD Los
RMP (mV)	−69.4±2.8	−59.5±3.9	−63.4±4.6	−67.6±1.6
APA (mV)	83.9±3.3	76.3±5.5	75.3±7.7	78.8±2.9
MaxNegSlope (mV/s)	2722±144	1707±180*,#	2624±133	2252±172
Triangulation (ms)	32.5±2.8	55.7±10.1*,#,†	32.2±2.1	26.7±2.1
APD30 - 1000 (ms)	16.5±0.6	20.7±1.7	15.2±2.6	21.4±1.9
APD30 - 800 (ms)	16.9±0.6	20.9±1.7	15.2±2.9	21.7±1.7
APD30 - 500 (ms)	17.5±0.6	21.2±1.3	14.6±2.9	22.5±1.6
APD30 - 300 (ms)	18.0±0.8	22.0±1.4	15.7±3.2	23.4±1.8
APD50 - 1000 (ms)	23.1±0.7	31.3±2.4	20.1±2.9	30.5±2.2
APD50 - 800 (ms)	23.4±0.7	30.5±2.4	22.4±3.6	29.6±1.9
APD50 - 500 (ms)	24.5±0.8	31.7±2.1	21.8±3.5	30.7±1.9
APD50 - 300 (ms)	25.3±1.1	32.3±2.2	23.8±3.7	31.9±2.1
APD90 - 1000 (ms)	52.4±2.6	81.7±10.8*,#,†	49.2±4.6	53.5±3.5
APD90 - 800 (ms)	52.3±2.5	79.3±7.9*,#,†	53.7±5.8	54.1±4.1
APD90 - 500 (ms)	54.1±3.0	73.6±5.1*,#,†	52.2±5.0	54.8±3.9
APD90 - 300 (ms)	56.1±2.7	75.4±5.7*	57.4±6.1	55.9±4.1

RMP: Resting membrane potential; APA: Action potential amplitude; APD: Action potential duration. The results are expressed as mean ± SEM; n = 6 (CTR), n = 5 (BRD), n = 4 (CTR Los), n = 5 (BRD Los). Statistical significance: * P<0.05 *vs.* CTR, #P<0.05 *vs.* CTR Los, †P<0.05 *vs.* BRD Los.

**Table 2:** Action potential parameters recorded in right endocardial ventricle preparation.

Variables	Groups			
	CTR	BRD	CTR Los	BRD Los
RMP (mV)	−61.4±1.0	−64.2±2.9	−60.7±2.8	−70.1±1.8
APA (mV)	76.3±2.2	81.1±5.1	70.3±5.5	88.0±3.0
MaxNegSlope (mV/s)	3822±258	3152±274	3691±445	4456±607
Triangulation (ms)	27.0±3.1	30.2±5.3	23.1±2.8	21.4±1.3
APD_30_ - 1000 (ms)	8.6±0.7	11.7±1.4	7.6±1.3	10.4±1.6
APD_30_ - 800 (ms)	9.1±0.5	12.0±0.5	7.3±1.2	11.1±1.6
APD_30_ - 500 (ms)	10.6±1.0	12.1±1.1	8.1±1.4	11.5±1.7
APD_30_ - 300 (ms)	10.9±0.6	14.2±1.9	8.8±1.3	12.4±1.9
APD_50_ - 1000 (ms)	13.3±0.9	17.2±2.0	11.3±1.9	14.6±2.2
APD_50_ - 800 (ms)	13.5±0.9	17.5±1.0	11.1±1.7	15.3±2.2
APD_50_ - 500 (ms)	16.0±1.7	17.7±1.7	12.2±2.0	16.1±2.3
APD_50_ - 300 (ms)	16.1±1.0	20.6±2.9	13.32±1.7	17.1±2.5
APD_90_ - 1000 (ms)	35.4±2.7	44.6±6.7	32.3±4.1	33.8±3.0
APD_90_ - 800 (ms)	35.3±2.9	45.7±4.8	31.8±4.0	34.8±2.8
APD_90_ - 500 (ms)	39.8±3.1	46.3±5.1	34.1±4.5	35.9±2.8
APD_90_ - 300 (ms)	37.8±2.4	49.2±6.8	36.7±3.4	37.7±3.3

RMP: Resting membrane potential. APA: Action potential amplitude; APD: Action potential duration. The results are expressed as mean ± SEM; n = 6 (CTR), n = 5 (BRD), n = 4 (CTR Los), n = 5 (BRD Los). No statistical differences were found among groups.

**Figure 8:**
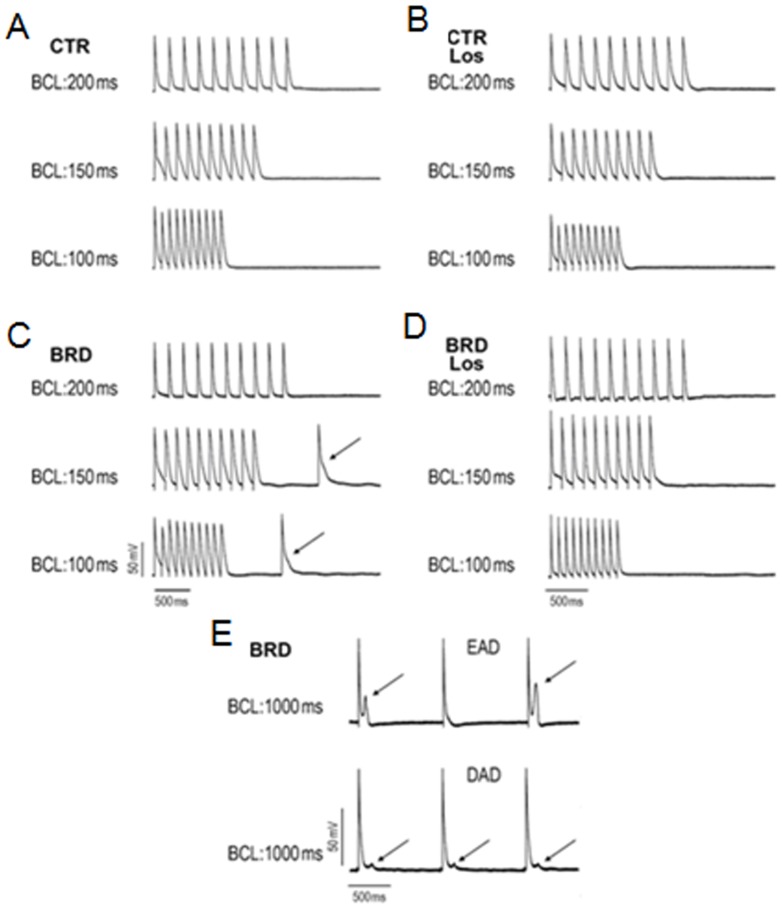
Triggered activity, early after depolarization (EAD) and delayed after depolarization (DAD), was induced by chronic BRD intake. (A–D) Representative action potential traces after a train of 10 beats at BCLs of 200, 150 and 100 ms followed by a pause in the left ventricle of all studied groups. (C) Representative action potential traces from the BRD group show rate-dependent triggered activity during a pause (arrows) after a train of 10 beats at BCLs of 150 and 100 ms followed by a pause protocol. (D) Los prevented the appearance of BRD-induced triggered activity. (E) In right ventricle BRD induced late-phase 3 EAD and DAD at BCL 1000 ms (arrows).

From the above results emerged the hypothesis that triggered activity, early after depolarization (EAD) and/or delayed after depolarization (DAD) could be present. To test this, trains of 10 beats at basic cycle lengths (BCLs) of 200, 150, and 100 ms followed by a pause were applied (Figure 8A–D). The most remarkable observation was the presence, in the BRD group, of a rate-dependent triggered activity during the pause in four out of five left ventricle endocardial preparations at BCLs of 150 ms and 100 ms (representatives in Figure 8C), also prevented by Los (Figure 8D) and not observed in the right ventricle (not shown). Late phase 3 EAD and DAD appeared spontaneously in two out of five right ventricle endocardial preparations only in the BRD group at a BCL of 1000 ms (Figure 8E).

### Contrasting effects on ouabain-resistant Na^+^-ATPase and (Na^+^+K^+^)ATPase activities

**Figure 9:**
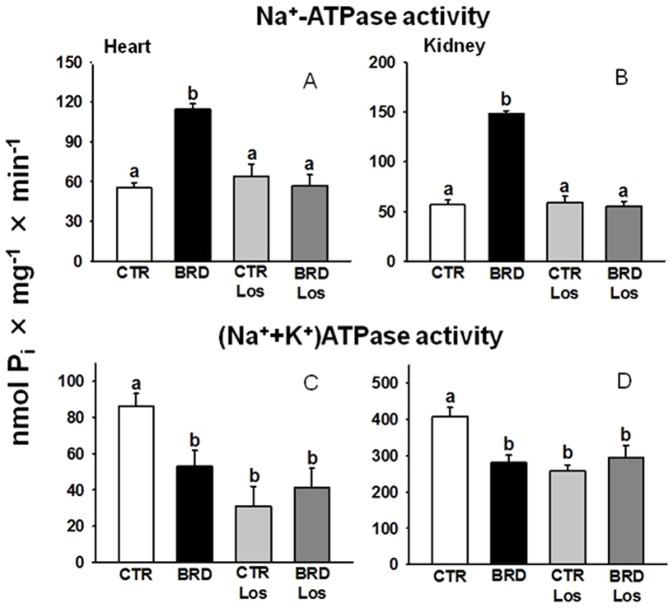
The increment of ouabain-resistant Na^+^-ATPase and the decrease of (Na^+^+K^+^)ATPase activities were similar in cardiomyocytes and renal proximal tubule cells of BRD rats, but the pumps were differentially modulated by Los. Upper: ouabain-insensitive Na^+^-ATPase (A, heart, n = 5; B, kidney, n = 5). Lower: (Na^+^+K^+^)ATPase (C, heart, n = 5; D, kidney, n = 5). Histograms show mean ± SEM. Different lowercase letters above the bars indicate statistically different mean values within the corresponding panel (P<0.05), assessed by one-way ANOVA followed by Tukey test for multiple comparisons.

Expansion of plasma volume, and the rich constellation of signs of cardiac remodeling, were then associated with dysfunctions in the active transport of Na^+^ (among others not considered in the present study). This plausible view is demonstrated in Figure 9. Chronic dietary restriction affected both Na^+^ active transporters similarly in left heart cardiomyocytes and renal proximal tubule cells, although the effect differed depending on the pump. There was a huge activation (more than 100%) of ouabain-resistant Na^+^-ATPase activity in cardiac and renal membranes, which was no longer hyperactive in the BRD rats treated with Los (Figure 9A and 9B). Administration of Los to the CTR rats did not modify the Na^+^-ATPase. (Na^+^+K^+^)ATPase activity decreased in BRD animals (Figure 9C and 9D) but the decrease was not modified by Los. In the CTR rats, chronic administration of Los also led to a comparable degree of inhibition.

### Altered density of Ang II receptors in membranes from left heart cardiomyocytes and proximal cells

**Figure 10:**
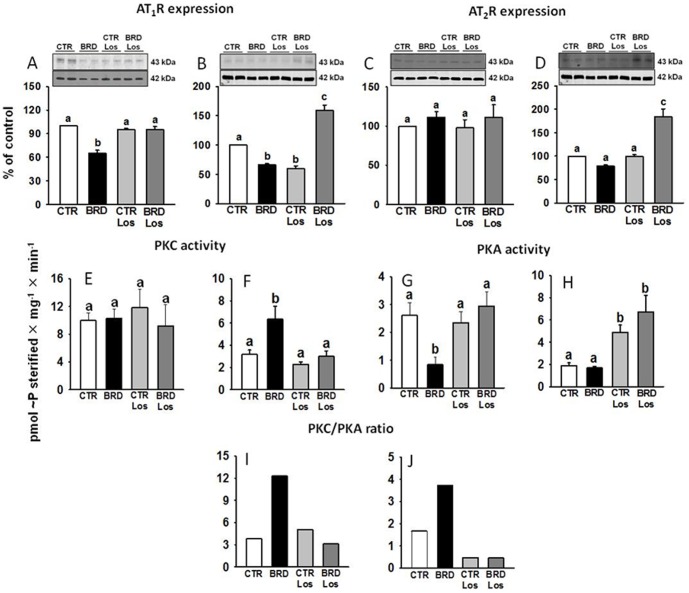
Chronic BRD intake altered Ang II receptor density in membranes and PKC and PKA activities. (A–D) AT_1_R and AT_2_R density. Upper panels: representative immunostainings (duplicates for each experimental condition) and densitometric representations (lower panels) of 7–12 experiments in duplicate corrected for protein loading (β-actin immunosignals in the corresponding lane, middle panels), which were carried out using different membrane preparations (left panels: heart; right panels: kidney). Different lowercase letters above the bars indicate statistically different mean values within the corresponding panel, assessed by one-way ANOVA followed by Tukey test for multiple comparisons. P values for AT_1_R comparisons: P<0.001 (BRD against the other three groups in heart, P = 0.607–1.000 when the other groups were compared among them); P<0.001 (BRD and CTR Los against the other two groups in kidney, P<0.001 BRD Los against the other three groups, P = 0.811 BRD versus CTR Los). P values for AT_2_R comparisons: P = 0.686 (heart, where differences among the four groups were not found and Tukey test was not carried out); P<0.001 (BRD against the other three groups in kidney, P = 0.317–1.000 when the other groups were compared among them). (E–J) PKC and PKA activities (n = 5–7), and PKC/PKA ratio (left panels: heart; right panels: kidney). Different lowercase letters above the bars indicate statistically different mean values within the corresponding panel, also assessed by one-way ANOVA followed by Tukey test.

AT_1_R and AT_2_R are the first components of a complex and interactive kinase-mediated signaling network that culminates in modulation of the renal Na^+^-ATPase by Ang II [40], [41]. Owing to the striking effect of chronic undernutrition on this pump and its reversal by Los, investigation of this network was started by examining AT_1_R and AT_2_R densities in cardiac and renal membranes. Expression of AT_1_R was decreased in the cardiomyocytes and proximal tubule cells from BRD rats (Figure 10A and 10B), thus confirming that they are relevant targets in cardiorenal dysfunction resulting from chronic undernutrition. However, the picture was not identical for AT_2_R or for the influence of Los. AT_2_R remained unmodified in cardiomyocytes (Figure 10 C) and in tubules from the BRD and CTR Los groups (Figure 10D). Los promoted an accentuated upregulation of both classes of receptors in the kidneys of rats submitted to BRD and downregulation of AT_1_R from the CTR group (Figure 10B and 10D). In cardiomyocytes, downregulation of AT_1_R was cancelled by Los (Figure 10A).

### Effects on PKC and PKA: imbalance in the PKC/PKA ratio

This scrutiny of the effect of the BRD diet on Ang II signaling pathways was followed by an investigation of PKC and PKA activities. PKC activity in heart membranes was not affected in all experimental groups (Figure 10E); conversely, BRD led to a significant increase of PKC (about 100%) in kidney membranes, which returned to control values in the Los-treated animals (Figure 10F). Contrasting effects on PKA activities in heart and kidney were also revealed (Figure 10G and 10H). While BRD strongly downregulated cardiac PKA activity, the diet induced no change in this kinase in the kidney. Moreover, the set of experiments with Los-treated animals revealed that the AT_1_R antagonist: (i) prevented the downregulation of cardiac PKA, (ii) had no effect on heart membranes from the CTR group, and (iii) upregulated PKA activity in kidney membranes from both CTR and BRD rats (Figure 10G and 10H). Since an imbalance between these two kinases is associated with alterations in renal active Na^+^ transporters promoted by perinatal programming with the same diet [8], we determined the ratio between them. This imbalance is clearly seen in Figure 10I and 10J: BRD strongly increased the PKC/PKA ratio in both organs, an effect that was reversed by Los.

### The MAPK/ERK1/2 network participates in the chronic undernutrition-induced activation of Na^+^-ATPase in cardiomyocytes and proximal tubule cells

**Figure 11:**
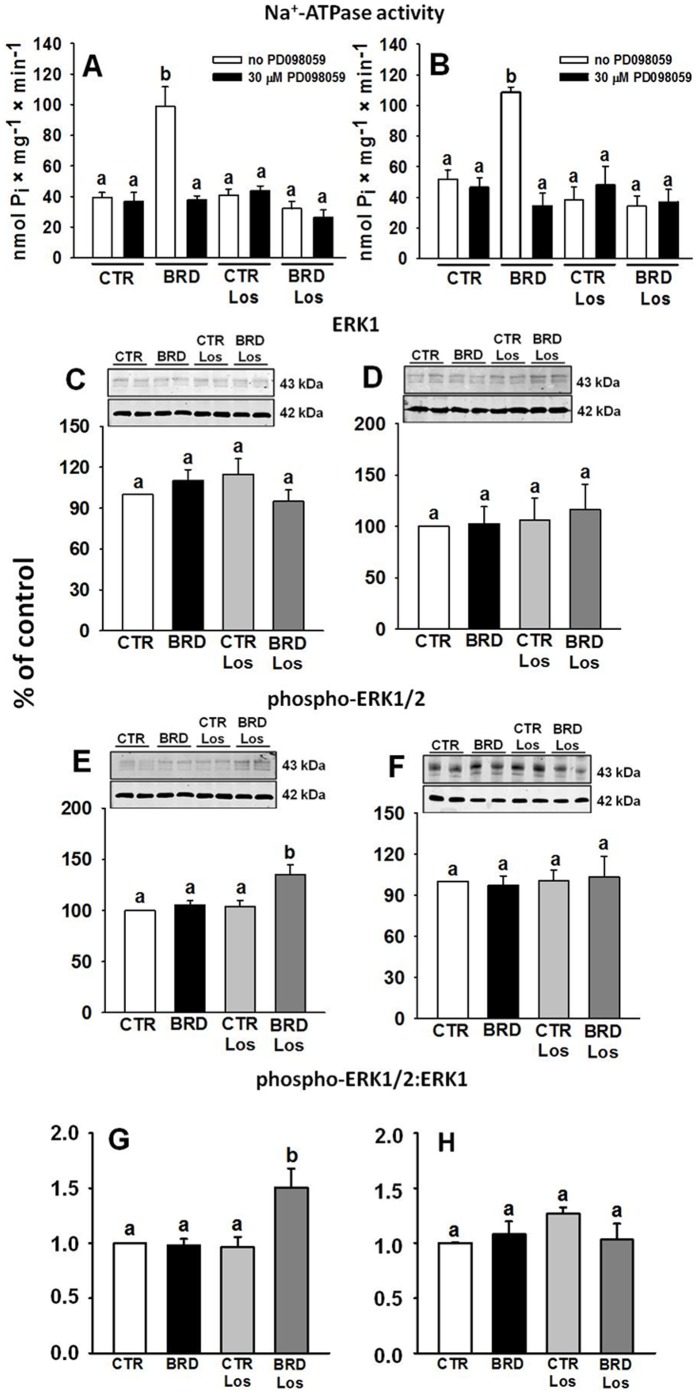
Na^+^-ATPase activity and MAPK pathway in heart (left panels) and kidney (right panels). (A, B) Na^+^-ATPase activity was measured in the four experimental groups in the absence or presence of 30 µM PD098059, as indicated. Results are mean ± SEM (n = 5) in assays carried out using different membrane preparations. (C, D) Representative immunoblottings of ERK1 in duplicate (upper panels), β-actin loading-controls for each blotting (middle panels) and densitometric representations (n = 8–10) (lower panels). (E, F) Representative immunoblottings of phospho-ERK1/2 in duplicate (upper panels), β-actin loading controls for each blotting (middle panels) and densitometric representations (n = 8–10) (lower panels). (G, H) phospho-ERK1/2∶ERK1 ratio (n = 8–10). Each phospho-ERK1/2∶ERK1 ratio value was calculated using the corresponding densitometric value obtained from the same lane. Different lowercase letters above the bars indicate statistically different mean values within the corresponding panel, assessed by one-way ANOVA followed by Tukey test. P values for ERK1 comparisons: P = 0.302 and 0.968 (heart and kidney, respectively, where no statistical differences were found among the four groups and Tukey test was not carried out). P values for phospho-ERK1/2 comparisons: P = 0.001–0.007 (heart, BRD Los against the other three groups, P = 0.928–0.999 for the comparisons among the other groups); P = 0.928 (kidney, where no statistical differences were found among the four groups and Tukey test was not carried out). P values for phospho-ERK1/2∶ERK1 ratio comparisons: P = 0.004–0.007 (heart, BRD Los against the other four groups, P = 0.995–0.999 for the comparisons among the other groups); P = 0.977 (kidney, no differences among the four groups).

Recently, Gildea et al., demonstrated crosstalk between the AT_1_R and the MAPK/ERK1/2 pathway in proximal tubules [42].To elaborate the view that other mechanisms could be involved in the modifications of the Na^+^ pumps, we first investigated the effect of PD098059, a specific inhibitor of MAPK. The enormous increase in Na^+^-ATPase activity in cardiomyocytes and in proximal tubule cell membranes from BRD rats was completely cancelled when PD098059 was added to the reaction medium (Figure 11A and 11B). The inhibitor had no effect on the activity measured in the other three groups, evidence of influence on a pathway that was selectively affected by BRD in rats not treated with Los. The influence of the inhibitor upon the (Na^+^+K^+^)ATPase was not assessed because, in contrast to the Na^+^-ATPase, it needs key cytosolic Ang II-linked regulatory components [43] that are not retained in the purified plasma membranes. The extent to which the expression of ERK1 and phospho-ERK1/2 is constitutive was compared among the four groups (Figure 11C–F). The most striking finding was the upregulation of phospho-ERK1/2 in the BRD group treated with Los, in both heart and renal membranes, with increase and preservation of the phospho-ERK1/2∶ERK1 ratio in heart and kidney, respectively (Figure 11G and 11H).

## Discussion

### Long-term general impact of dietary restriction

In this study we present evidence that chronic protein, mineral and vitamin restriction, a systemic pathological state that affects about one billion people across the world, simultaneously affects heart and kidney functions in young adult rats, leading to heart hypertrophy, electric cardiac remodeling, expanded plasma volume and the onset of hypertension in young adult rats. The driving hypothesis of this study was that chronic undernutrition affects cardiac and renal function at an early age (90 days). Moreover, the deficient diet, which mimics those used in vast impoverished regions of the world, is too drastic, such that survival decreases after 100 days of age. The lighter body weight – and the increase in systolic pressure – was accompanied by a phenotype resembling that found in marked and stable hypertension, including cachexia and severe lethargy, as seen by others [17]. This phenotype, therefore, can be considered a predictive marker for a reduced lifespan under huge chronic undernutrition that severely impacts on the cardio-renal axis. The hypothesis is that blocking AT_1_R increases lifespan (currently under investigation). As a central finding, the present report addresses unknown mechanisms regarding the modulation during protein restriction, and the activities in heart and kidney of the recently cloned and purified ouabain-insensitive Na^+^-ATPase [32], [44], the machinery responsible for fine-tuning Na^+^ transport across the plasma membrane [45].

### Role of Na^+^-ATPase: possible mechanisms underlying electric cardiac remodeling

In the kidney, the huge increase in ouabain-insensitive Na^+^-ATPase activity can explain the expanded plasma volume in chronically undernourished rats *via* an increase in Na^+^ flux from the lumen to the renal interstitium, leading to simultaneous increments in heart rate, systolic pressure, and heart weight/body weight ratio. To date, no reports have indicated the physiological influence of the ouabain-resistant Na^+^ in cardiomyocytes, which was described in rabbit cardiac sarcolemma several years ago [46]. On the basis of the findings presented here, it can be proposed that restoration of the normal level of this pumping activity by Los, together with the direct influence of the drug mentioned above, could make a key contribution to the prevention of cardiac electric remodeling in the BRD Los rats, thus counteracting the proarrhythmia risk consequent on (Na^+^+K^+^)ATPase downregulation.

The mechanisms described above involving the two modes of active Na^+^ flux can, therefore, help to explain mechanistically why BRD rats present electrocardiograph modifications, increasing the risk of cardiac arrhythmias and sudden death. Moreover, the risk of cardiac electrical disturbances could be partially explained by the presence of beat-dependent triggered activities and of EAD and DAD events. A plausible mechanism for the genesis of EAD and increased APD could involve the increment of I_Na_ with no increase in peak I_Na_, a disorder that is associated with abnormal cellular Na^+^ handling, as recently suggested for rat cardiomyocytes [47].

### Participation of RAS and kinase-mediated phosphorylations

Blocking of AT_1_R completely abolishes the activation of Na^+^-ATPase provoked by BRD, thus confirming the hypothesis of a central effect on the RAS, alterations in which could arise from a direct influence of the low-protein BRD – and possibly of an altered plasma amino acid composition – on tissue Ang II. Protein restriction during gestation programmed the downregulation of signaling components of the RAS with effects on blood pressure [7], [48], and this seems also to be the case for chronic protein deprivation through eating BRD after weaning. It has been demonstrated that RAS is important in the pathological heart hypertrophy [49], [50] that is counteracted by blocking AT_1_R [51] with simultaneous prevention of cardiac electric remodeling [52]. The view that kidney receptors are associated with heart receptors in the cardiac structural and electric remodeling receives further support from the observation that elimination of AT_1_R in mice reduces cardiac hypertrophy and the risk of hypertension [49], and also from the observation that primary renal dysfunction in rats is associated with an augmented risk of congestive cardiac failure in simultaneous alterations that constitute a cardiorenal syndrome [53].

The consequences of BRD, which are similar in heart and kidney in terms of Na^+^ pumping activities and can explain the integration of pathophysiological events, are partially distinct in terms of Ang II receptors and protein kinases. The different profiles of Ang II receptors expression and PKC and PKA activities can shed light on their tissue-specific participation in the alterations of electric activity (heart) and fluid handling (kidney). The decrease in AT_1_R combined with an imbalance between PKC and PKA (increased PKC/PKA ratio in BRD rats) could contribute to both upregulation of Na^+^-ATPase activity and decreased (Na^+^+K^+^)ATPase activity in both organs. It has been demonstrated that normal balance between these kinases is essential for the modulation of Na^+^-ATPase [40], [41]. Thus, the Los-induced decrease in the PKC/PKA ratio could contribute to restoration of the Na^+^-ATPase activity upregulated in BRD rats by the simultaneous increase in PKC and decrease in PKA. The Los-induced recovery of the PKC/PKA ratio in cardiomyocytes could be also a key mechanism in the prevention of electric cardiac remodeling. Increased expression of both AT_1_R and AT_2_R in renal membranes of the BRD group treated with Los could be explained by modifications in the association of the two classes of receptors that perturb their mutual signaling exchange [54] or by stimulation of intracellular partners that facilitates their insertion into the membranes, as proposed for AT_2_R [55]. Possibly, the Los-induced decreased abundance of renal AT_1_R in CTR rats relies on the same complex and not completely elucidated pathway interactions in proximal kidney tubules [42].

### Involvement of the MAPK/ERK1/2 pathway

**Figure 12:**
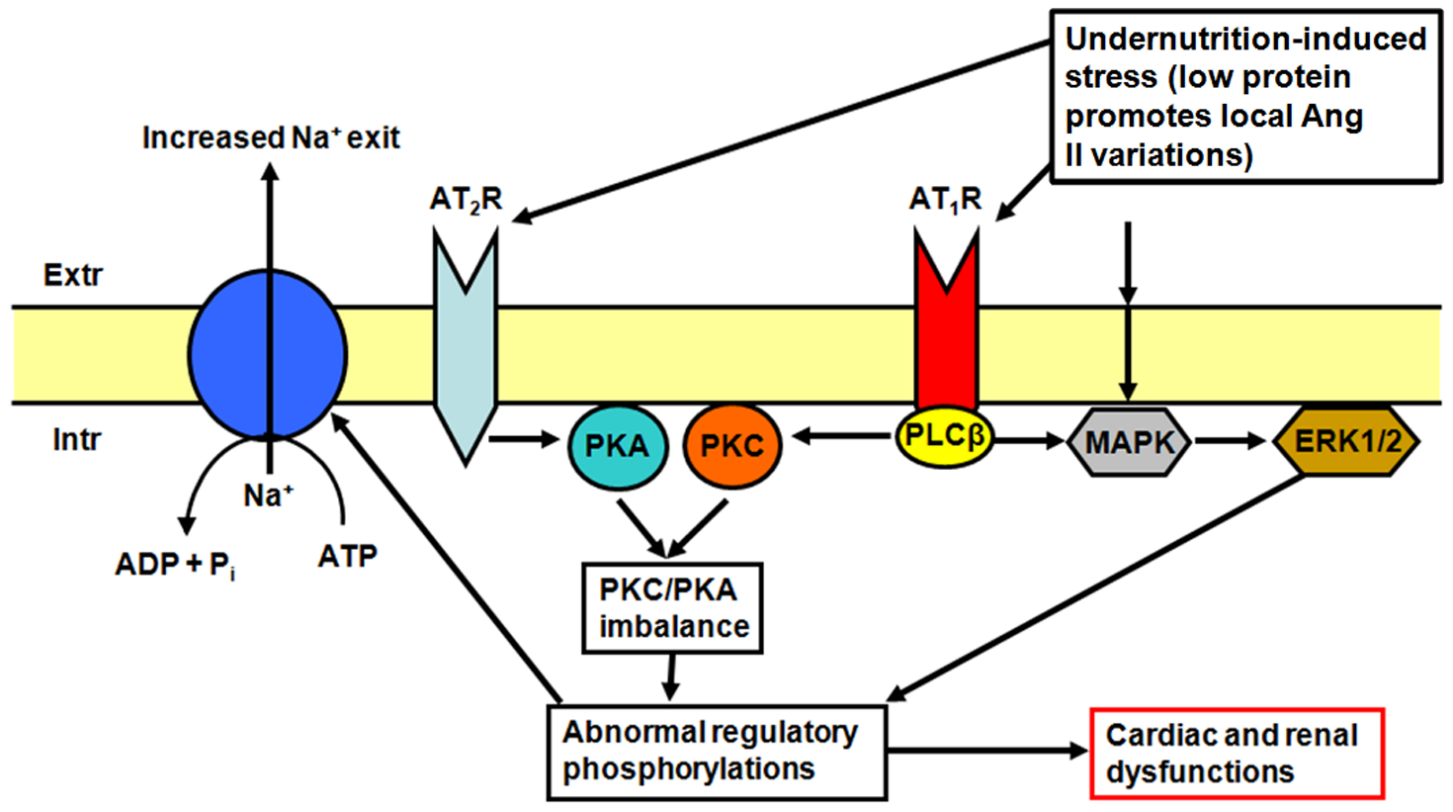
Proposed interactions among Ang II receptors, PKC/PKA, MAPK and ERK1/2 in heart and kidney from BRD rats, ultimately targeting the ouabain-insensitive Na^+^-ATPase and therefore the fine tuning of Na^+^ extrusion. Extracellular-induced stress (low protein and altered local Ang II levels) can induce wrong signaling in the branches linked to AT_1_R and AT_2_R as well as increase of MAPK/ERK1/2 turnover. The resulting imbalance (increase) in PKC activity/PKA activity ratio, together with modified ERK1/2 activity, would result in abnormal regulatory phosphorylation(s) of the ouabain-insensitive Na^+^-ATPase and culminate in the accelerated Los- and PD98059-sensitive turnover of the pump (Figure 9A and 9B). The representation also indicates a possible link between AT_1_R and MAPK/ERK1/2 according to ref. [42].

The MAPK/ERK1/2 signaling routes seem to be crucial for regulating cardiac physiological and pathological events [56]–[60]. In the present study, the combined effects of Los administration, the recovery of control values of ouabain-insensitive Na^+^-ATPase when the MAPK inhibitor PD098059 was present in the assays, and the normal values of Na^+^-ATPase activity from non-Los BRD rats when MAPK was blocked, help to elucidate – at least in part – the interrelated mechanisms on which rest the simultaneous cardiac and renal alterations induced by BRD. The proposal of crosstalk between the Ang II and MAPK/ERK1/2 cascades finds support in the observations that (i) Ang II phosphorylates cardiac ERK1/2 *in vivo* [60]; (ii) Ang II phosphorylates ERK1/2 from proximal tubule cells *in vitro* [61] in events that involve Ang II receptors [62]; (iii) Ang II receptor signaling modulates the phosphorylation of ERK1/2 in renal proximal tubules [42]. Since there was no additive action on Na^+^-ATPase when AT_1_R are blocked or when MAPK is blocked, it is likely that the wrong signals induced by BRD interact with different kinases and, ultimately, converge on that key target for Na^+^-handling. These proposed interactions are outlined in Figure 12.

It is possible that undernutrition-induced stress [7], [8], [18], [22], [63] evokes abnormal constitutive modifications in MAPK leading directly to modifications in ERK1/2 activity, irrespective of signals from AT_1_R. As in the case of Ang II receptors densities, the modifications in the phospho-ERK1/2∶ERK1 ratio in heart and kidney from the BRD Los group exhibit different images (increase and preservation, respectively), thus pointing to a tissue-specific outcome of protein restriction, despite the final common effect on Na^+^-ATPase. Finally, the lack of influence of PD098059 upon Na^+^-ATPase in Los-treated rats is consistent with the proposal that PKC exerts a permissive action in the catalysis by phospho-ERK1/2 [64].

Although this model could provide a step towards elucidating the complex cardiorenal alterations induced by chronic protein restriction associated with other dietary deficiencies, it is clearly far from complete. Ang II in serum and left ventricle/kidney cortical tissues was not measured. However, two observations support the link between protein restriction and altered local Ang II levels. First, Ceravolo et al. [63] demonstrated that intrauterine undernourished rats presented at 16 weeks with moderate hypertension, and increased mesenteric vascular reactivity *in vivo*-*in situ* to Ang II, with both alterations being normalized by Los. These findings strongly support an increased local activation of the AT_1_R-mediated pathway, as proposed in Figure 12. Second, we recently demonstrated using the same diet that adult offspring from dams undernourished during pregnancy gradually develop hypertension with age, a process that is concomitant with an increase in Na^+^-ATPase activity and an augmented number of Ang II-positive cells in the kidney cortex [65]. Thus, even though the window of exposure to the deficient diet was different in this parallel study (gestation), the increased Ang II local levels in proximal tubule cells seems indicative that protein restriction at different periods of development could be a key stress factor that activates the AT_1_R-mediated pathway in different tissues.

It is also plausible that other axes of plasma volume regulation – and therefore, of arterial pressure – are affected by dietary restriction, especially in the kidney and its proximal tubules, as focused upon in this study. Cornock and et al. [66] showed that pregnant rats fed a low-protein diet downregulated aquaporin 2 – the water channel in the apical membrane of collecting ducts – in conjunction with a significantly lower expression of AT_2_R. These observations point to an additional effect of undernutrition on the handling of renal fluid in distal segments and possibly the disruption of the delicate balance between proximal and distal transport processes.

Finally, the important alterations in plasma amino acids and in the overall nutritional status could also have important implications for the observed alterations in Na^+^ pumping activities. The huge increases of L-Serine, L-Threonine, L-Histidine and L-Alanine that accompany that of L-Glutamine – the key amino acid for renal cortex metabolism – with simultaneous decrease of L-Valine and L-Leucine, suggest that chronic BRD administration led to tissue proteolysis in an attempt to compensate for the poor quality of the ingested protein. The mirror images L-Serine/D-Serine and of L-Alanine/D-Alanine also indicates BRD-induced racemization. The alterations in these plasma amino acids could be sensed by intracellular receptors involved in adaptations to the availability of nutrients, such as mTOR, as recently demonstrated [67], with subsequent effects on signaling pathways and pumps. Moreover, signals coming from the altered levels of (Na^+^-K^+^)ATPase – possibly as a result of the altered plasma amino acids – can in turn influence the kinase pathways, including the mTOR pathway, in a cell-specific manner, as demonstrated in [68].
